# Close access to health care as a bridge overcoming disparities in thyroid cancer

**DOI:** 10.1007/s12020-026-04618-3

**Published:** 2026-05-02

**Authors:** Larisa Aizikovich, Uri Yoel, Merav Frenkel, Liroy Caracucli, Ronit Harris, Oded Cohen

**Affiliations:** 1 Faculty of Health, Ben Gurion University of the Negev, Negev, Israel; 2 Department of Endocrinology, Soroka Medical Center, Negev, Israel; 3 Department of Otolaryngology, Head and Neck Surgery, Samson Assuta Ashdod, Ashdod, Israel

**Keywords:** Differentiated Thyroid Cancer (DTC), Papillary thyroid cancer (PTC), Follicular thyroid cancer (FTC), Negev population, Ethnicity, Bedouin, Disparity

## Abstract

**Introduction:**

Studies occasionally demonstrate various ethnic disparities regarding differentiated thyroid cancer (DTC) characteristics and outcomes. The impact of public health care and close access to care has been studied scarcely. With a unique minority group within the hospital region, this case control study aims to investigate this further.

**Methods:**

A retrospective cohort study of patients with DTC diagnosis who followed-up at a tertiary medical center between 2013 and 2024. The patients were categorized into Minority (study group) or non-minority (control group) and were reviewed for DTC characteristics at presentation and outcomes including histopathology, risk of structural disease recurrence, response to initial treatment, and mortality and risk group according to the 2015 ATA guidelines [1].

**Results:**

A total of 316 patients were included, of whom 100 (31.6%) constituted the minority group. Two hundred forty-two patients (77%) were females. The minority group was diagnosed at a younger age compared to non-minority group (41 ± 15 vs. 53 ± 15 years, *p* < 0.001) and had higher rates of female patients (82% vs. 74%, *p* = 0.12, respectively). The median follow-up of the cohort was 4.41 (2.25–7.21) years. The minority cohort presented with significantly larger tumors (median 20 mm vs. 15 mm, *p* = 0.042) and a significantly higher prevalence of multifocality (46% vs. 31%, *p* = 0.03) compared to the non-minority cohort. Conversely, the rate of extrathyroidal extension (ETE) was lower in the minority cohort, although this did not reach statistical significance (21% vs. 32%, *p* = 0.10, respectively). DTC characteristics did not demonstrate significant differences regarding LN or distant metastasis. While the extent of surgery, ATA risk classification and response to initial therapy were comparable between the groups, the minority cohort required significantly more additional post-operative interventions (20% vs. 11%, *p* = 0.039). During the study period, 21 (6.6%) patients died, including 6 (1.9%) disease-specific deaths, with no between-group differences in all-cause or disease-specific mortality.

**Conclusions:**

Our findings suggest that a good access to non-insurance based, public healthcare may reduce thyroid cancer disparities, which was reported repeatedly up to date. Health system structure and language barriers may play a role in disparities attributed to ethnicity.

**Supplementary Information:**

The online version contains supplementary material available at 10.1007/s12020-026-04618-3.

## Introduction

 Over the past few decades, differentiated thyroid cancer (DTC) prevalence has been on the rise globally, primarily driven by an increase in papillary thyroid cancer (PTC) diagnoses. As incidence increases, concerns regarding quality of care and disparities are becoming a key interest for health providers [2–7].

Various factors contributing to the disparities seen in DTC patients have been recognized and established. Patients with higher socioeconomic status (SES) or better insurance often face overdiagnosis of small, clinically insignificant PTC due to better access to advanced diagnostics [3, 8]. These disparities also extend to the presentation of the disease, as studies indicate that patients who are socioeconomically disadvantaged, have no insurance, residing in rural areas, or belonging to minoritized racial and ethnic groups tend to be diagnosed at later stages [4].

Most studies on DTC disparities have been conducted in private insurance settings, which can affect various factors, including diagnosis rates and disease severity. After a literature review, to the best of our knowledge, no article has addressed disparities in a funded public health-system.

Bedouins Arabs are considered a unique minority group who are routinely referred to our institution, based on the proximity of their settlements to the facility. They are characterized.

by unique tribal norms and rules including high rates of consanguineous marriage and are known for its low SES, exampled by temporary housing, lack of constant electricity and more [9, 10]. Despite a close physical distance to a tertiary center, several studies have shown reduced utility of health care [5, 11].

This setting allowed us to investigate disparities in low income, culturally- separated minority, yet with a very high access to a tertiary center with excellent level of care, if wishing so. Furthermore, as public the health policy in our country remains social rather than mostly private, insurance-based one, we anticipate some disparities in the diagnosis and treatment of DTC may occur but to a lesser extent of private insurance-based system. Moreover, comparison of the histologic factors would help to suggest possible impact of cultural background, either genetic or environmental, on DTC subtypes and clinical presentation.

Herein we aim to evaluate differences in the clinical, histopathological characteristics, treatment modalities and response to treatment among DTC minority patients, compared with all other, non-minority DTC patients attending our institution.

## Methods

### Study design and population

This retrospective cohort study was approved in advance by our Institutional Review Board (protocol code SOR-0003-18, approval date 27 March 2018). The requirement for informed consent was waived due to the retrospective design. We analyzed the individual medical records of all patients with post thyroidectomy diagnosis of DTC, who were followed at our center facilities between December 2013 and March 2024. All patients of 18 years or older at DTC diagnosis were included. Exclusion criteria included patients with missing or incomplete pertinent medical records and inoperable patients and patients with less than 6 months following diagnosis. Since the purpose of the study was to evaluate DTC alone, all non-DTC cancers, including patients with poorly differentiated, medullary and anaplastic thyroid cancer were excluded as well. The study’s cohort was then divided based on ethnicity into minority (Bedouin, Arabs, the study group) and the non-minority populations, represented mainly by Jewish ethnicity.

The primary outcome of this study is the distribution of the disease stage at the time of diagnosis and histopathological features among DTC patients. The secondary outcomes include the risk of structural recurrence and response to initial treatment (Based on the American Thyroid Association guidelines [ATA], see below [1]), and prognosis (overall survival and disease specific survival).

Data collection involved the extraction and summary of the individual patient medical record, allowing access to imaging, pathological reports, lab reports, admissions and follow-up visits, thus providing a comprehensive view of the patients’ health.

All the medical records were reviewed, and the data collected included the following: patients’ age at diagnosis, sex, ethnicity (minority vs. non-minority population), BMI and comorbidities. Regarding comorbidities, we extracted data on the presence or absence of specific diseases: diabetes, pre-diabetes, hypertension and ischemic heart disease prior to surgery. In addition, we reviewed the following laboratory tests conducted prior the surgery: creatinine(mg/dL), TSH(µIU/mL) and the chosen treatment modality including the type of surgical procedure - total thyroidectomy, thyroid lobectomy and whether the patient received radioiodine therapy. Data regarding the patient’s response to the therapy was classified according to the 2015 ATA management guidelines [1]. We have also extracted pathological reports, detailing the tumor’s pathological characteristics (size, sub-type, single vs. two-lobe involvement, extrathyroidal extension invasion (Categorized into three levels: No ETE, Microscopic ETE and Gross ETE), vascular invasion (Categorized as No, Yes or Multiple), positive lymph nodes, bilateral lateral lymph nodes, extra nodal extension, distal metastasis).

### Statistical analyses

Continuous variables are presented as mean ± SD or median (range), as appropriate. Categorical variables are summarized as frequencies and percentages. Between-group comparisons by ethnicity were performed using t tests for normally distributed continuous variables and the Wilcoxon rank-sum test for non-normally distributed variables. Categorical variables were compared using Pearson’s chi-square test or Fisher’s exact test when expected cell counts were < 5. A two-sided *P* ≤ 0.05 was considered statistically significant.

Statistical analyses were performed on the overall study cohort (*N* = 316) and subsequently on a sub-cohort excluding microcarcinomas (tumors > 1 cm, *n* = 237) in order to assess whether inclusion or exclusion of microcarcinomas may confound the results of the study.

All statistical analyses were conducted using R software (R Core Team, 2021; R: A Language and Environment for Statistical Computing; R Foundation for Statistical Computing, Vienna, Austria; https://www.R-project.org/).

## Results

**Fig. 1:**
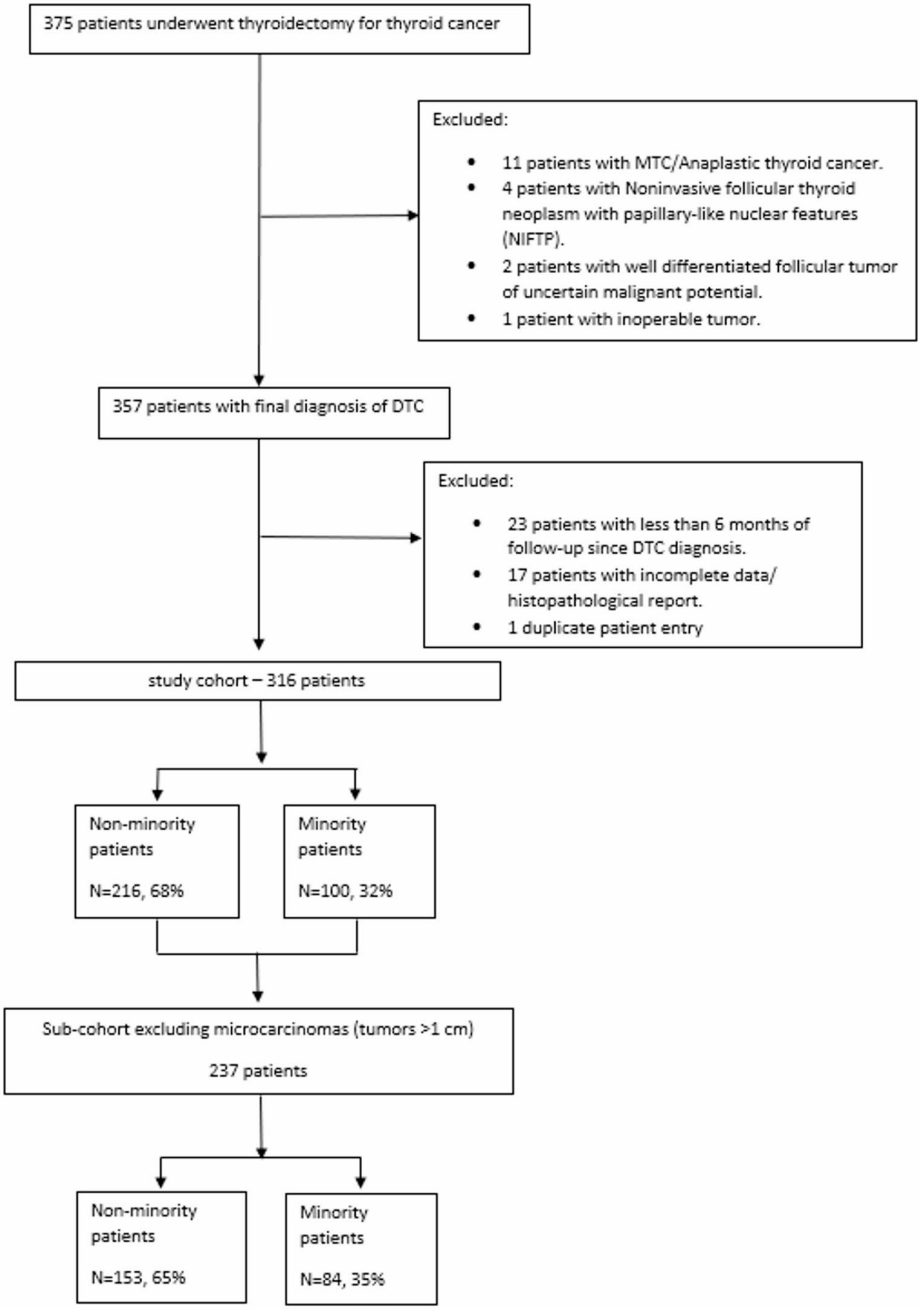
Study Flowchart

Overall, a total of 375 patients underwent partial or complete thyroid surgery during the study period. After exclusion, 316 patients were included in the overall cohort, of whom 237 patients had a tumor size > 1 cm, as displayed in Fig. 1.

Patients’ demographics and pre-operative characteristics are presented in Table 1. A hundred patients (31.6%) were included in the minority (study) cohort and 216 (68.4%) in the non-minority cohort. The majority of patients were females (77%, 242/316), with a higher proportion in the minority cohort compared to the non-minority cohort (82% vs. 74%, *p* = 0.12). The mean age at diagnosis was 49 ± 16 years. The minority cohort patients were diagnosed at a significantly younger age compared to the non-minority cohort (41 ± 15 vs. 53 ± 15 years, *p* < 0.001, respectively). The median TSH was lower among the minority cohort compared to the non-minority cohort (1.35 [IQR 0.90–2.22] vs. 1.70 [IQR 1.12–2.61] µIU/ml, *p* = 0.010), yet within the reference range, and the absolute difference (0.35–0.37 µIU/mL) is unlikely to be clinically meaningful.

**Table 1:** Baseline demographic, clinical and biochemical characteristics of patients who underwent thyroidectomy with final DTC histology report, according to their ethnicity group

	Overall	Non-Minority	Minority (Bedouin Arabs)	*p*-value^2^
	*N* = 316^*1*^	*N* = 216^*1*^	*N* = 100^*1*^	
**Gender (Female)**	242 (77%)	160 (74%)	82 (82%)	0.12
**Age at diagnosis**	49 ± 16	53 ± 15	41 ± 15	< 0.001
**Hypertension**	84 (27%)	67 (31%)	17 (17%)	0.008
**Pre-diabetes/ diabetes-mellitus**	92 (29%)	67 (31%)	25 (25%)	0.3
**Ischemic heart disease**	17 (5.4%)	16 (7.4%)	1 (1.0%)	0.019
**BMI**	28.0 ± 5.2	27.9 ± 5.0	28.1 ± 5.5	0.8
**Creatinine** ^**Ψ**^	0.71 (0.60–0.86)	0.74 (0.64–0.89)	0.63 (0.54–0.73)	< 0.001
**TSH**^**Ψ**^, **µIU/mL**	1.77 (1.14–2.76)	1.88 (1.18–2.82)	1.51 (1.01–2.54)	0.019

^*1*^ n (%); Mean ± SD; Median (Q1-Q3)

^*2*^ Pearson’s Chi-squared test; Welch Two Sample t-test; Wilcoxon rank sum test^Ψ^last result prior to thyroid surgery. DTC- differentiated thyroid carcinoma, BMI- body mass index(body weight [Kg] divided by the square of height[meters]), N-number, SD- standard deviation, TSH – thyroid stimulating hormone

Total thyroidectomy was performed on 185 patients (59%), with comparable rates in both minority and non-minority cohorts (56% vs. 60%, *p* = 0.7). Completion of thyroidectomy following a lobectomy was performed in 14% of cases (17% vs. 13%, *p* = 0.4).

Post-operative histopathological characteristics of both groups are presented in Table 2. The minority cohort presented with significantly larger tumors (median 20 mm vs. 15 mm, *p* = 0.042) and a significantly higher rate of multifocality compared to the non-minority cohort (46% vs. 31%, *p* = 0.03, respectively). The rate of extrathyroidal extension (ETE) (microscopic + gross) was comparable (21% vs. 32%, *p* = 0.10, respectively). no additional statistically significant differences in histopathological characteristics were observed between the groups (Table 2).

**Table 2:** Histopathological features following thyroidectomy of patients diagnosed with DTC, according to their ethnicity group

	Overall	Non-Minority	Minority (Bedouin Arabs)	*p*-value^2^
	*N* = 316^1^	*N* = 216^1^	*N* = 100^1^	
**Tumor size**				0.042
<=1	78 (25%)	62 (29%)	16 (16%)	
1–2	128 (41%)	87 (40%)	41 (41%)	
2–4	78 (25%)	49 (23%)	29 (29%)	
>4	31 (9.8%)	17 (7.9%)	14 (14%)	
**Multifocality**				0.03
No	174 (55%)	129 (60%)	45 (45%)	
Two foci in one lobe	45 (14%)	25 (12%)	20 (20%)	
Both lobes	97 (31%)	62 (29%)	35 (35%)	
**Extrathyroidal extension invasion**				0.1
No	227 (72%)	148 (69%)	79 (79%)	
Microscopic	67 (21%)	53 (25%)	14 (14%)	
Gross	22 (7.0%)	15 (6.9%)	7 (7.0%)	
**Vascular invasion**				0.6
No	256 (81%)	176 (81%)	80 (80%)	
Yes	41 (13%)	29 (13%)	12 (12%)	
Multiple	19 (6.0%)	11 (5.1%)	8 (8.0%)	
**positive LN**				0.3
N1a central	52 (16.5%)	36 (16.7%)	16 (16%)	
N1b lateral	49 (15.5%)	38 (17.6%)	11 (11%)	
None	215 (68%)	142 (65.7%)	73 (731%)	
**Bilateral lateral LN**	15 (4.7%)	11 (5.1%)	4 (4.0%)	0.8
**Extra nodal extension**	39 (12%)	30 (14%)	9 (9.0%)	0.2
**Distal metastasis**	13 (4.1%)	10 (4.6%)	3 (3.0%)	0.8

^*1*^ n (%)

^*2*^ Pearson’s Chi-squared test; Fisher’s exact test^§^ Response to treatment as was documented in the last follow-up. DTC- differentiated thyroid carcinoma, N-number, SD- standard deviation, LN- lymph node/s

Treatment modalities and follow-up data of patients with DTC are presented in Table 3. The median follow-up was 4.41 (2.25–7.21) years, with a significantly longer follow-up period in the minority cohort compared to the non-minority cohort (5.10 [2.58–8.13] vs. 4.18 [1.98–6.39] years, *p* = 0.015).

**Table 3:** Treatment modalities and follow-up data of patients with DTC, stratified by ethnicity

	Overall	Non-Minority	Minority (Bedouin Arabs)	*p*-value^2^
	*N* = 316^1^	*N* = 216^1^	*N* = 100^1^	
**All-cause mortality**	21 (6.6%)	17 (7.9%)	4 (4.0%)	0.2
**Disease specific mortality**	6 (1.9%)	4 (1.9%)	2 (2.0%)	> 0.9
**RAI treatment**	167 (53%)	111 (51%)	56 (56%)	0.4
**Risk assessment**				0.5
Low	164 (52%)	110 (52%)	54 (54%)	
Intermediate	108 (35%)	72 (34%)	36 (36%)	
High	41 (13%)	31 (15%)	10 (10%)	
**Response to treatment** ^**§**^				0.9
Excellent	231 (73%)	159 (74%)	72 (72%)	
Biochemical incomplete	41 (13%)	26 (12%)	15 (15%)	
Structural incomplete	26 (8.2%)	19 (8.8%)	7 (7.0%)	
Indeterminate	18 (5.7%)	12 (5.6%)	6 (6.0%)	
**Intervention**				0.3
No intervention	285 (90%)	193 (89%)	92 (93%)	
More than one intervention	30 (9.5%)	23 (11%)	7 (7.1%)	

^§^ Response to treatment as was documented in the last follow-up. DTC- differentiated thyroid carcinoma, N-number, SD- standard deviation, RAI- radioactive iodine therapy

^*1*^ n (%); Median (Q1-Q3)

^*2*^  Pearson’s Chi-squared test; Fisher’s exact test; Wilcoxon rank sum test

While no significant difference was found in radioactive iodine (RAI) treatment (*p* = 0.5), the minority cohort received significantly more additional post-operative interventions compared to the non-minority cohort (20% vs. 11%, *p* = 0.039). The distribution of ATA risk categories differed significantly between the two groups (*p* = 0.019), with low risk being notably more common in the minority cohort (52% vs. 40%). Response to initial therapy did not differ between the groups, with an excellent response being the most prevalent in both cohorts (77% vs. 71%, *p* = 0.4). Mortality rates remained low and comparable between the groups, with an all-cause mortality of 6% (3.4% vs. 7.6%, *p* = 0.2) and a disease-specific mortality of 2% (1.7% vs. 2.0%, *p* > 0.9).

## Analysis of Patients with Tumors > 1 cm

Overall, 237 patients were included in this analysis, 84 (35%) in the study group and 153 (65%) in the control. The analysis is presented in Supplement Tables 1, 2 and 3, respective to Tables 1, 2 and 3. In general, all major outcomes were comparable between the two cohorts (with or without microcarcinomas). Significant differences found between the study group and non-minority groups of patients with tumors > 1 cm included the following findings:

Among patients with tumors > 1 cm, both multifocality and tumor size showed no significant difference between the two groups (*p* = 0.4, *p* = 0.2, respectively, *n* = 237), with comparable tumor size distribution (tumors 1–4 cm: minority 83% vs. non-minority 89% and tumors > 4 cm: 17% vs. 11%, respectively, *p* = 0.2).

In addition, there were no significant differences in post-operative treatments (RAI or additional interventions) between the two cohorts (*p* = 0.5 and 0.081, respectively).

The distribution in ATA risk categories showed borderline significance between the two groups (*p* = 0.092), with low risk being the most common in both groups (42%).

Of note, similar to the overall cohort, response to initial therapy did not differ between the groups, with an excellent response being the most prevalent in both cohorts (71% vs. 69%, *p* = 0.9, respectively).

## Discussion

The purpose of this study was to evaluate the impact of ethnicity on thyroid cancer presentation and outcomes in a region with good access to healthcare and a unique minority group, which was shown to demonstrate disparities in different health aspects [4, 5]. While our hypothesis was that certain differences would be found in tumor presentation, treatment strategies, or response to treatment, our results have shown no significant difference between the minority and non-minority cohorts.

The question whether ethnicity may be associated with differences in DTC presentation, severity and outcomes remains unsettled. The management of DTC varies across different countries and affected by health systems, insurance models and the resources of each-factors that complicate comparisons. Studies have repeatedly addressed the association between ethnicity and minority related disparities in DTC management and outcomes. Across many studies conducted in the USA, ethnic and racial minority groups consistently presented with more advance disease at presentation and received less guideline-based concordant care, in contrast to the results in our study. Gillis et al. reviewed multiple U.S. studies and demonstrated that racial and ethnic minority patients present with significantly more advanced disease; showing that different minorities had 36%-89% higher odds of metastatic disease at presentation [6]. Ginzberg et al. showed that insurance-based disparities in DTC treatment persist in the USA even after the 2015 ATA de-escalation guidelines. Patients who had Medicaid insurance - a public health insurance program for low-income individuals, had presented with more advanced disease compared to privately insured patients including larger tumors, regional metastases and less likely to undergo appropriate surgery [5]. A possible explanation for the conflict between our study and those of the aforementioned studies is the public health system in our country, which provides free health services for the population, especially in cases of cancer, in which regulatory restriction of district and choice of care do not apply. Therefore, it may be that the health system structure plays a larger role than ethnicity itself.

Zmijewski et al. conducted a systematic review focusing on racial disparities in DTC outcomes and found evidence of more advanced local disease in non-white patients. Among 7,221 patients, ETE was significantly more common in black and in hispanic compared to white patients [7]. In contrast with Zmijewski et al., our study demonstrated significantly lower ETE rates in minority group compared to the control group (24% vs. 40%, *p* = 0.032 for patients with tumors > 1 cm). These alleged contradicting results may imply again that access to care and health system structure may play the bigger part when discussing DTC-related disparities. The early detection of the disease due to our equal access to medical evaluation, in contrast to minorities in USA who often reach specialist care later and by this increase the chances that their tumor advances to ETE. Another explanation could be the younger age in which minority population was diagnosed compare with the aforementioned study [12]. The younger minority population tend to be more integrated, less traditional and has less language barriers, which, in turn, may increase the communication quality and may reduce delays linked to misunderstanding or under use of medical services.

Chen et al. demonstrated that socioeconomic instability, the absence of private insurance and language barrier delay time to treatment. They claim that limited language knowledge is likely contributes to the TC related care disparities observed among the Hispanic and the Asian patients in USA [2]. This finding can serve as another possible explanation to the lack of differences found in this current study. While culturally separate, it is very rare for our minority group patients to be unable to express their concerns in their mother language, as primary doctors speaks both languages and translation is very accessible either by family or staff. Therefore, it is possible that language may play a crucial role in its ability to bridge disparities among minorities with DTC.

Though did not demonstrate any significant differences regarding TC, in previous studies conducted in our region, this minority group has demonstrated significant disparities in other domains; Jaffer et al. demonstrated the socioeconomic disadvantages the Bedouins living in rural residencies are facing, such as limited transportation, high unemployment and poor living conditions-which have been linked to poorer health indicators and chronic disease burden [12]. Plakht et al. documented disparities in the field of cardiovascular outcomes, showing that Bedouin patients, especially women, have worse prognosis following an acute myocardial infraction [11]. There are several possible explanations that come to mind. To begin with, the setting of DTC management differs from chronic illnesses; chronic illnesses require continuous care: regular clinic visits, adherence to medication regiment and self-monitoring in cases of DM or HTN, which may be affected by those disparities over time and lead to poorer outcomes. Whereas DTC care is episodic, shorter and protocol driven, which is easier for adherence. More importantly, the diagnosis of cancer carries substantial stress among patients and family, driving to pursue care promptly. Finally, it is possible that relatively short follow-up and small sample of the cohort resulted in the differences between our study and findings found in the US.

Our study has several limitations. First, its retrospective nature, relying on existing records was a major limitation. Second, our cohort size was relatively small, resulting in underpowered sub-group analyzes. Additional factors that may contribute to racial/ethnic disparities in DTC including socioeconomic status and actual place of residency were not captured in our database due to lack of data, yet it well known that this minority is within the very low percentile of our country’s socioeconomic scale.

## Conclusion

Our findings suggest that good access to non-insurance based, public healthcare may reduce DTC disparities, which was reported repeatedly up to date. Health system structure and language barriers may play a role in disparities attributed to ethnicity.

## Electronic Supplementary Material

Below is the link to the electronic supplementary material.

Supplementary Material 1

## Data Availability

The datasets generated and analyzed during the current study are not publicly available due to patient privacy and hospital regulations, but are available from the corresponding author on reasonable request.
