# Supplementary material for: Close access to health care as a bridge overcoming disparities in thyroid cancer
Source: Endocrine. 2026 May 2;91(1):148. doi: 10.1007/s12020-026-04618-3 (PMC13135005; doi:10.1007/s12020-026-04618-3)
Supplement: Supplementary file 1 — Supplementary Material 1 [file 12020_2026_4618_MOESM1_ESM.docx]

Supplement tables

*Table 1s – Baseline demographic, clinical and biochemical characteristics of patients who underwent thyroidectomy with final DTC histology report,* and tumor >1 cm *according to their ethnicity group.*

|  | **Overall** | **Non-Minority** | **Minority (Bedouin Arabs)** | **p-value***^2^* |
| --- | --- | --- | --- | --- |
|  | N = 237*^1^* | N = 153*^1^* | N = 84*^1^* |  |
| **Gender (Female)** | 173 (73%) | 104 (68%) | 69 (82%) | 0.019 |
| **Age at diagnosis** | 50 ± 17 | 54 ± 16 | 42 ± 15 | <0.001 |
| **Hypertension** | 73 (31%) | 58 (38%) | 15 (18%) | 0.001 |
| **Pre-diabetes/ diabetes-mellitus** | 70 (30%) | 49 (32%) | 21 (25%) | 0.3 |
| **Ischemic heart disease** | 15 (6.3%) | 14 (9.2%) | 1 (1.2%) | 0.016 |
| **BMI** | 28.3 ± 5.2 | 28.5 ± 5.1 | 28.0 ± 5.3 | 0.5 |
| **Creatinine^Ψ^** | 0.72 (0.60-0.88) | 0.77 (0.66-0.94) | 0.63 (0.54-0.73) | <0.001 |
| **TSH^Ψ^, µIU/mL** | 1.71 (1.16-2.77) | 1.85 (1.23-2.89) | 1.50 (1.01-2.52) | 0.021 |
| *^1^* n (%); Mean ± SD; Median (Q1-Q3) | | | | |
| *^2^* Pearson’s Chi-squared test; Welch Two Sample t-test; Wilcoxon rank sum test  ^Ψ^last result prior to thyroid surgery. Abbreviations: DTC- differentiated thyroid carcinoma, BMI- body mass index(body weight [Kg] divided by the square of height[meters]), N-number, SD- standard deviation, TSH – thyroid stimulating hormone. | | | | |

*Table 2s - Histopathological features following thyroidectomy of patients diagnosed with DTC,* and tumor >1 cm*, according to their ethnicity group.*

|  | **Overall** | **Non-Minority** | **Minority**  **(Bedouin Arabs)** | **p-value***^2^* |
| --- | --- | --- | --- | --- |
|  | N = 237*^1^* | N = 153*^1^* | N = 84*^1^* |  |
| **Tumor size** |  |  |  | 0.2 |
| 1-4 | 206 (87%) | 136 (89%) | 70 (83%) |  |
| >4 | 31 (13%) | 17 (11%) | 14 (17%) |  |
| **Multifocality** |  |  |  | 0.4 |
| No | 121 (51%) | 83 (54%) | 38 (45%) |  |
| Two foci in one lobe | 32 (14%) | 18 (12%) | 14 (17) % |  |
| Both lobes | 84 (35%) | 52 (34%) | 32 (38%) |  |
| **Extrathyroidal extension invasion** |  |  |  | 0.032 |
| No | 156 (66%) | 92 (60%) | 64 (76%) |  |
| Microscopic | 59 (25%) | 46 (30%) | 13 (15%) |  |
| Gross | 22 (9.3%) | 15 (9.8%) | 7 (8.3%) |  |
| **Vascular invasion** |  |  |  | ‘  0.4 |
| No | 182 (77%) | 116 (76%) | 66 (79%) |  |
| Yes | 37 (16%) | 27 (18%) | 10 (12%) |  |
| Multiple | 18 (7.6%) | 10 (6.5%) | 8 (9.5%) |  |
| **positive LN** |  |  |  | 0.8 |
| N1a central | 47 (52%) | 34 (51%) | 13 (54%) |  |
| N1b lateral | 44 (48%) | 33 (49%) | 11 (46%) |  |
| **Bilateral lateral LN** | 14 (5.9%) | 10 (6.5%) | 4 (4.8%) | 0.8 |
| **Extra nodal extension** | 38 (16%) | 29 (19%) | 9 (11%) | 0.1 |
| **Distal metastasis** | 13 (5.5%) | 10 (6.5%) | 3 (3.6%) | 0.4 |
| *^1^* n (%) | | | | |
| *^2^* Pearson’s Chi-squared test; Fisher’s exact test  ^§^ Response to treatment as was documented in the last follow-up. Abbreviations: DTC- differentiated thyroid carcinoma, N-number, SD- standard deviation, LN- lymph node/s. | | | | |

*Table 3s –* Treatment modalities and follow-up data of patients with DTC, and tumor >1 cm, stratified by ethnicity.

|  | **Overall** | **Non-Minority** | **Minority**  **(Bedouin Arabs)** | **p-value***^2^* |
| --- | --- | --- | --- | --- |
|  | N = 237*^1^* | N = 153*^1^* | N = 84*^1^* |  |
| **All-cause mortality** | 19 (8.0%) | 15 (9.8%) | 4 (4.8%) | 0.2 |
| **Disease specific mortality** | 6 (2.5%) | 4 (2.6%) | 2 (2.4%) | >0.9 |
| **RAI treatment** | 156 (66%) | 103 (67%) | 53 (63%) | 0.5 |
| **Risk assessment** |  |  |  | 0.092 |
| Low | 99 (42%) | 57 (38%) | 42 (50%) |  |
| Intermediate | 97 (41%) | 64 (42%) | 33 (39%) |  |
| High | 39 (17%) | 30 (20%) | 9 (11%) |  |
| **Response to treatment^§^** |  |  |  | 0.9 |
| Excellent | 165 (70%) | 105 (69%) | 60 (71%) |  |
| Biochemical incomplete | 38 (16%) | 24 (16%) | 14 (17%) |  |
| Structural incomplete | 22 (9.3 %) | 16 (10%) | 6 (7.1%) |  |
| Indeterminate | 12 (5.1%) | 8 (5.2%) | 4 (4.8%) |  |
| **Intervention** |  |  |  | 0.081 |
| No intervention | 207 (88%) | 130 (85%) | 77 (93%) |  |
| More than one intervention | 29 (12%) | 23 (15%) | 6 (7.2%) |  |
| *^1^* n (%); Median (Q1-Q3) | | | | |
| *^2^*  Pearson’s Chi-squared test; Fisher’s exact test; Wilcoxon rank sum test | | | | |
| ^§^ Response to treatment as was documented in the last follow-up. Abbreviations: DTC- differentiated thyroid carcinoma, N-number, SD- standard deviation, RAI- radioactive iodine therapy. | | | | |
